# EHMTI-0082. Use of IV steroids in cluster headache

**DOI:** 10.1186/1129-2377-15-S1-C11

**Published:** 2014-09-18

**Authors:** L Savi, L Pinessi, C Condello

**Affiliations:** 1 Headache Center, Città Della Salute e Della Scienza, Torino, Italy

## Background

Steroids are very effective in aborting episodes of Cluster Headache (CH), both orally and intravenously (IV). Anyway, formal studies to assess this efficacy are lacking. A placebo-controlled study with oral steroid has received approval and is currently under development. To our knowledge no study focusing to IV steroid use for blocking acute episodes has been published yet.

## Aim

Tto evaluate retrospectively a case series of patients with CH episodes treated with infusion of corticosteroid.

## Methods

A series of 16 consecutive patients observed in our Headache Centre in the last 3 years for acute episodes of CH unresponsive to standard management (short tapering of oral steroids and Verapamil per os). All of them were male, of age between 37 and 69 years old.

They underwent rescue treatment with IV methylprednisolone 500 mg for 3 or for 5 days, followed by slow tapering per os. One half of the patients received Verapamil per os, too, in fast titration and to the maximum dose of 720 mg/day split in three doses.

## Results

By the end of the IV steroid, 13 out of 16 patients were free from attacks (81.2%). Of the three unresponsive patients, one was diagnosed with a possible somatoform disturb. The two others received concomitantly maximum doses of Verapamil, to no avail.

## Conclusion

IV steroids are effective in blocking episodes of CH. In the acute phase improvement does not seem correlated to addition of Verapamil, which is likely more useful in preventing reapparition of attacks shortly after steroid discontinuation.

No conflict of interest.

